# The causal effects of artificial intelligence use on metacognition, engagement, and knowledge transfer in educational contexts

**DOI:** 10.3389/fpsyg.2026.1811065

**Published:** 2026-05-19

**Authors:** Jian Li

**Affiliations:** School of Innovation and Entrepreneurship, Harbin University, Harbin, Heilongjiang, China

**Keywords:** artificial intelligence, causal inference, EdNet, engagement, knowledge transfer, learning analytics, metacognition, tutoring systems

## Abstract

**Introduction:**

Artificial intelligence applications in learning environments are becoming more common, but there is no clear causal evidence regarding the impact of AI-assisted tutoring on metacognition, engagement, and knowledge transfer.

**Methods:**

Using EdNet log data, AI use was operationalized as exposure to tutoring-system assistance events during learning episodes. Fixed effects models with temporal controls within learner and clustered inference were estimated. Monitoring proxies and control proxies were used to assess metacognition, time on task, activity intensity, persistence after errors, return probability, and spacing. Engagement, near transfer, topic-shift transfer, and delayed performance were assessed.

**Results:**

AI exposure increased monitoring behaviors (*β* = 0.028–0.047) and control behaviors (*β* = 0.036–0.061). Longer time on task (*β* = 0.083), higher activity intensity (*β* = 0.057), higher persistence after errors (*β* = 0.064), and higher return probability on the next day (*β* = 0.031) increased engagement, while shorter interval between activities (*β* = −0.018) decreased it. The strongest transfer gains were for near transfer (*β* = 0.052), topic-shift transfer (*β* = 0.021), and delayed performance (7 days) (*β* = 0.017).

**Discussion:**

AI-supported tutoring appears to strengthen metacognitive regulation and engagement, while broader transfer remains conditional on scaffolds that promote verification, retrieval, and abstraction.

## 1 Introduction

It has become part of everyday learning, with conversational systems that provide explanations on command and adaptive tutoring services that suggest practice sequences and provide instant feedback (Deng et al., 2024). However, the pace of adoption has exceeded our capacity to make plausible causal claims about how AI use redesigns fundamental learning activities. An emerging body of empirical evidence indicates that AI tools have the potential to enhance short-term performance and experience of learners in most activities, particularly by offering timely feedback and minimizing iteration and revision obstacles (Meyer et al., 2024). Simultaneously, there has been concern that AI may foster superficial thinking, overdependence, and a weak sense of competence, which is not generalizable to the absence of support (Melumad and Yun, 2025). This tension is supported by recent experimental evidence: learners using large language models to receive synthesized answers can be, in some cases, less knowledgeable than learners who must derive facts through slower, information-seeking processes, even when the content they are learning is similar (Bhattarai, 2025). In a similar vein, the literature on generative AI and feedback interaction within higher education also suggests that there is an essential boundary condition: advantages are contingent on how learners decipher and respond to AI feedback, such as whether to consider AI suggestions as a form of reflective cues or as a kind of replacement of thought (Mekheimer, 2025; Xu et al., 2025).

Despite this growing evidence, the subject matter still does not present a unified causal explanation of how the application of AI affects metacognition, engagement, and knowledge transfer as a unified whole. Most studies employ self-report, cross-sectional correlational designs, or brief interventions, primarily reporting outcome scores but not tracing the learning process that produced them (Wang and Fan, 2025). This gap is well known in the context of learning analytics: observational log data are plentiful, yet causal inference is considered desirable but not operational, with results subject to selection effects, time-dependent confounding, and vague definitions of treatment (Weidlich et al., 2022). Methodological advice has increasingly emphasized stating causal questions as effects, explicitly identifying assumptions, and designs that implement diagnostics and falsification tests, rather than relying on a proxy for predictive success as an indicator of causal validity (Dahabreh and Bibbins-Domingo, 2024). These arguments are particularly pressing in educational AI, since AI use is rarely unplanned: students decide when to request assistance, when to be offered advice, and when to tune out, and such decisions are closely tied to prior knowledge, motivation, and task complexity.

To address the gap noted above, this paper examines the causal relationship between AI use and three outcomes central to learning science and directly related to the special issue topic: metacognition, engagement, and knowledge transfer. We do not view metacognition as a highly introspective characteristic but rather as a set of observable regulatory behaviors evident in digital traces, including help seeking, verification, revisiting, and strategic switching under uncertainty. We conceptualize engagement as a dynamic behavioral process characterized by persistence, interspersal, patterns of returns, and in-session effort. We consider transfer to be the capacity to apply knowledge across items and contexts, which can be estimated by performance on new items within the same underlying skill and by successful transfer across skill changes. This framing aligns with recent research focusing on generative AI literacies and the agency of learners, in which self-regulation is posed as a factor between productive use and dependency (Anders and Speltz, 2025), and where the effects of AI on engagement and learning have been shown to differ depending on how students incorporate AI into their workflow (Granström and Oppi, 2025; Xu et al., 2025).

EdNet aligns well with this agenda because it provides the scale, behavioral richness, and temporal coverage necessary to relate AI-mediated learning interactions to process-level outcomes. The EdNet is a very large hierarchical set of interactions between students and the system that is gathered in an AI tutoring environment and contains not only correctness events but a broader range of learning behaviors and platform behavior, allowing analysis as well as analysis with respect to final scores and into the learning process itself (Choi et al., 2020). It is now acknowledged that the dataset has emerged as a fundamental reference point of knowledge tracing and student modeling studies, in which the aim is to track the dynamics of knowledge across sequences of interactions (Parker et al., 2025; Shen et al., 2024). In a broader sense, it is embedded in a mature ecosystem of open educational datasets and data analysis practices, which is well-suited to transparent and reproducible causal learning analytics, in combination with thoughtful design decisions and diagnostics (Mihaescu and Popescu, 2021).

Most significantly for causal questions, the longitudinal logs that EdNet provides enable us to treat treatment as time-localized AI-related exposure during learning episodes and to model outcomes as follow-up behavior and performance rather than a fixed endpoint. The structure enables modern causal designs that explicitly manipulate temporal ordering, leverage within-learner variation, and test identification assumptions through pre-trend checks, negative controls, and robustness specifications (Dahabreh and Bibbins-Domingo, 2024; Weidlich et al., 2022). Recent studies on learning analytics have highlighted that observational educational log data can be used to address causal questions, which are made explicit in the design, assumptions, and bias structure, especially in longitudinal contexts where repeated observations are made on the same learners. Educational technology log data have been studied before, and have demonstrated that fine-grained usage data can be causally analyzed, although also identifying frequent threats, including time-varying confounding, feedback effects, and practice effects. In that regard, our within-learner fixed-effects design adheres to a well-developed design-based rationale: it leverages repeated observations within learners to minimize bias from constant learner heterogeneity and subject the remaining identifying assumptions to scrutiny (Sales and Pane, 2021; Yhee and Park, 2026). By making metacognition and behavioral traces the foundation of our analysis, EdNet enables us to move beyond the broad assertion that AI is either helpful or harmful and instead to examine when AI is used to transform regulatory behaviors, when it creates or reduces persistence, and whether any short-term benefits of AI use are transferred. Thus, the paper will provide causal evidence that is not only methodologically sound but also practical, usable by educators and designers to support the positive application of AI in real-world learning settings. We employ this by using within-learner fixed-effects models that leverage longitudinal changes in AI exposure between repeated learning episodes for the same student. This identification design eliminates all time-varying learner traits (e.g., ability, motivation, background) and separates the causal influence of AI support and constant individual variations. The most critical identifying assumption is that, at least holding fixed learner effects and time-varying controls, the timing of a learner’s exposure to AI support is not systematic due to unobserved shocks that also influence the outcomes. We test this assumption using pre-trend, falsification, and overlap tests. There are three key contributions in this paper. First, it provides causal evidence on the influence of AI-assisted tutoring sessions on metacognition, engagement, and knowledge transfer, thereby filling an important gap in prior research, which has relied primarily on correlational or self-reported studies. Second, it models these constructs using behaviorally rooted proxies derived from learning traces of large-scale data, thereby enabling the study of metacognitive control and interaction processes in realistic AI-mediated learning settings. Third, it integrates these outcomes into a single causal framework that explains when AI use aids learning and when it poses a threat of substitution and overdependence.

**Figure 1:**
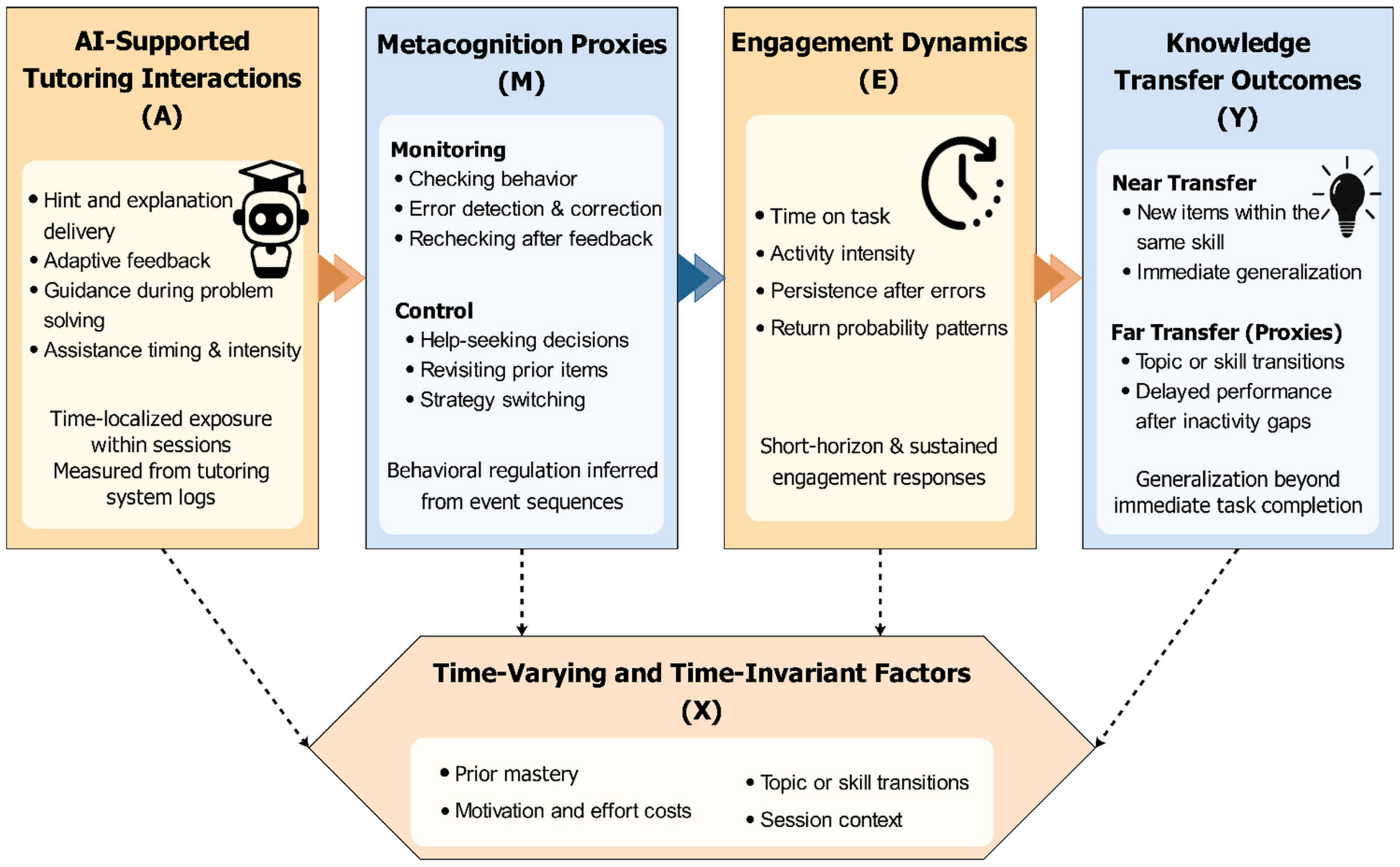
AI-supported tutoring interactions to metacognition proxies, engagement, and knowledge transfer.

As illustrated in Figure 1, the conceptual model links AI-mediated tutoring experiences to observable metacognitive proxies and involvement behaviors, which, in turn, influence knowledge transfer outcomes. Within the framework of this paper (Figure 1), the rest of it is structured accordingly: the following section elaborates theory-based hypotheses, the section that follows provides a description of the dataset and strategy of causing the identification, and the other sections develop empirical findings and strength analysis of the findings, and the paper closes by discussing theoretical implications and practical suggestions on how to design and apply educational AI systems.

The paper is organized as follows. Section 2 introduces the conceptual frameworks and hypotheses that connect the AI-based tutoring to metacognition, engagement, and knowledge transfer. The data source, the operational definitions, and the causal identification strategy are outlined in Section 3. Section 4 presents the empirical findings, including effects on metacognition, engagement, and transfer, and provides rigorous analyses. Section 5 talks about the findings, theoretical implications, practical guidance, and limitations. Section 6 concludes by providing future research directions.

## 2 Conceptual framework and hypotheses

Our conceptualization of the application of educational AI in EdNet is exposure to system-mediated assistance events within a learner’s problem-solving sequence, including requests and the receipt of hints, explanations, step-by-step instructions, or adaptive feedback. We will attempt to measure the effect of such exposure on the learning process rather than only on the final results. We adopt the traditional two-level perspective on metacognition, according to which monitoring generates information about prevailing knowledge bases, and control mobilizes that information to allocate effort and select strategies (Nelson, 1990; Nelson and Narens, 1994). AI support may function as a scaffold that enhances surveillance and control in this configuration, but may also operate as a substitute, thereby reducing autonomous regulation. We formalize the causal target in terms of potential outcomes. Let $A_{\mathrm{it}}\in \{0,1\}$ denote whether the learner i receives AI support at the time t within a session, and let $Y_{i,t+\Delta }(a)$ denote a downstream outcome measured after a lag Δ under exposure level a . The primary estimand is an average treatment effect, by using Equation (1):

$$\tau (\Delta )=E\;[Y_{i,t+\Delta }(1)-Y_{i,t+\Delta }(0)]$$ (1)

with secondary effects for heterogeneity $\tau (\Delta |X_{i})$ , which $X_{i}$ contains prior performance and activity charge. Because AI exposure and selection into support are time-varying and behaviorally relevant, we use explicit causal assumptions and graphical reasoning to disaggregate treatment effects from confounding, consistent with modern practice in causal inference (Berzuini et al., 2012).

### 2.1 AI-supported learning and metacognition in log data

Metacognition is operationalized as monitoring and control behaviors that can be inferred from event streams. Monitoring is manifested by signals that a learner is examining progress or uncertainty, including prediction-like behaviors (attempt confidence when available), checking behaviors, and error diagnosis, whereas control is manifested by choices that control cognition, including revising answers, switching strategies, seeking help, or terminating an attempt (Nelson, 1990; Nelson and Narens, 1994). In intelligent tutoring systems, help seeking is typically treated as a canonical example of metacognitive control: students may choose when to seek help, when to persevere, and when to continue despite feedback (Aleven et al., 2006; Roll et al., 2011). EdNet can be used especially well due to its ability to record the various types of actions other than correctness, and form behavioral proxies used to monitor and control behavior at a fine time scale (Choi et al., 2020). We denote metacognition proxy outcomes as $M_{\mathrm{it}}^{\mathrm{mon}}$ for monitoring behaviors and $M_{\mathrm{it}}^{\mathrm{ctl}}$ controlling behaviors. Representative monitoring proxy Representation Representative probability of verification behavior that follows an attempt, represented as the conditional probability of a verification action, given a successful response: $M_{\mathrm{it}}^{\mathrm{mon}}=\mathrm{Pr}({\text{Check}}_{\mathrm{it}}=1|{\text{Attempt}}_{\mathrm{it}}=1)$ . Other monitoring proxies are the rate of error-correction, which is $\mathrm{Pr}({\text{Correct}}_{i,t+1}=1|{\text{Wrong}}_{\mathrm{it}}=1,{\text{Feedback}}_{\mathrm{it}}=1)$ . For example, the probability of asking for help after an incorrect attempt is a control proxy, by using Equation (2):

$$M_{\mathrm{it}}^{\mathrm{ctl}}=\mathrm{Pr}(H_{\mathrm{it}}=1|{\text{wrong}}_{\mathrm{it}}=1)$$ (2)

or a revision proxy, which is determined as the probability of modifying the answer with the help of feedback. The main theoretical issue is whether AI support transforms the informativeness of monitoring and makes control more effective, or leads to more monitoring and, thus, weaker control over time (Aleven et al., 2006; Roll et al., 2011).

### 2.2 Engagement pathways in AI-mediated learning

It is the dynamic nature of engagement as a behavior that is directly measurable in log data and can be explained psychologically. Consistent with prevailing engagement frameworks, behavioral engagement comprises participation, persistence, and time investment, whereas cognitive engagement is characterized by effort and sustained attention (Fredricks et al., 2004; Kahu, 2013). In tutoring logs, we operationalize engagement in terms of quantifiable measures such as session length, attempt frequency, persistence after errors, and the interval between learning sessions on different days. For learner i , let $S_{\mathrm{id}}$ be the set of sessions on day d . A simple daily engagement intensity can be defined by using Equation (3):

$$E_{\mathrm{id}}=\sum_{s\in S_{\mathrm{id}}}{\mathrm{dur}}_{\text{is}}+\lambda \sum_{s\in S_{\mathrm{id}}}{\text{attempts}}_{\text{is}}$$ (3)

and spacing can be summarized using inter-session gaps $\Delta t_{\text{is}}=t_{i,s}-t_{i,s-1}$ and their distributional features. In Equation 3, λ is a tuning constant that is not negative, and it gives greater emphasis to the role of effort in the score on composite engagement intensity in relation to the length of the session. Our default setting of λ=1 assigns equal importance to both parts after each is found in its natural units (minutes and attempts, respectively). The direction and significance of the engagement effects were found to be robust to this choice by sensitivity analyses with λ∈{0.5,,1,,2} . AI assistance may increase engagement by reducing frustration and enabling progress, yet engagement may decrease when learners become passive consumers of solutions rather than active creators of meaning (Aleven et al., 2006).

**Figure 2:**
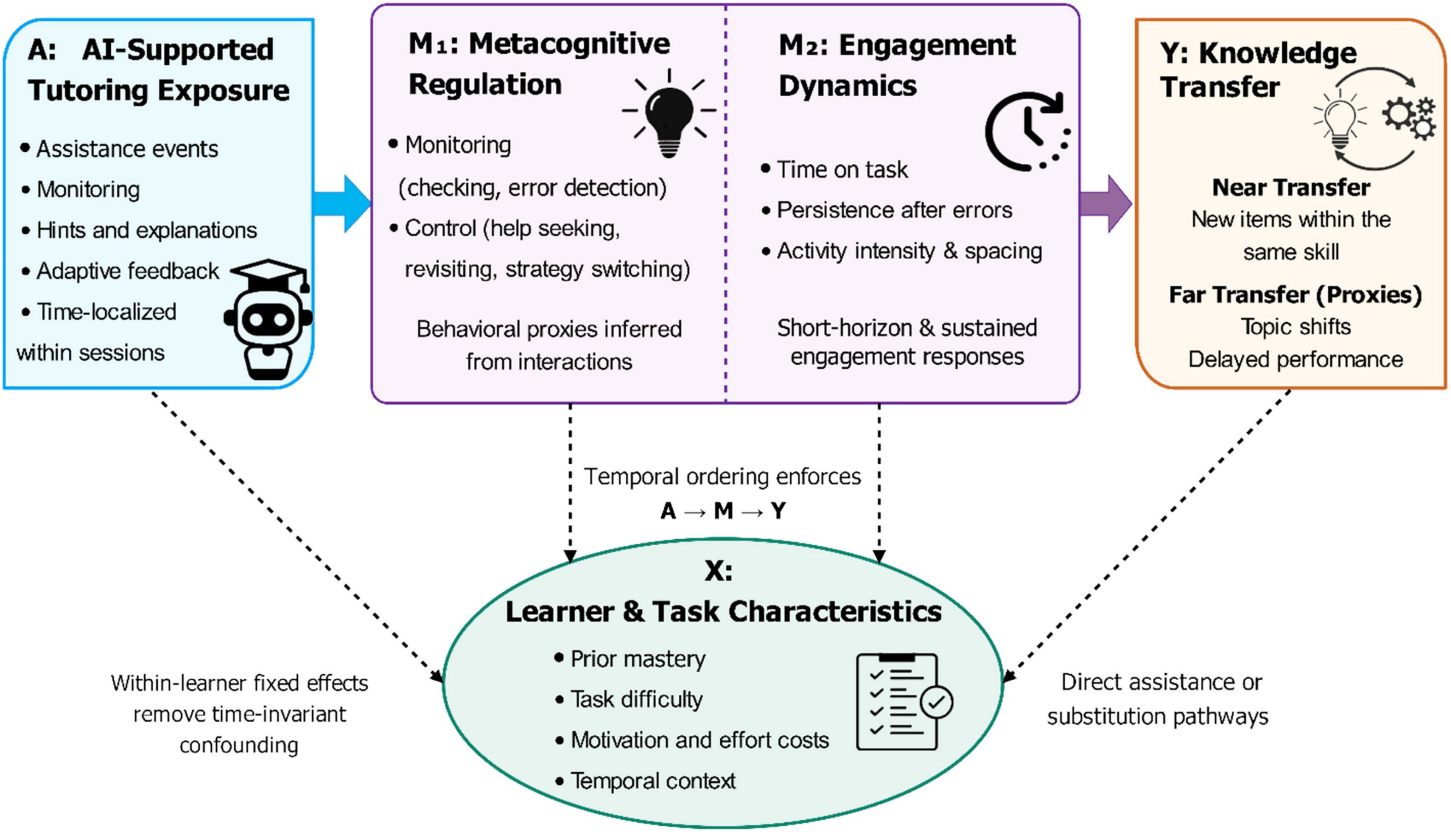
Directed causal graph and mechanism pathways.

The directed causal graph in Figure 2 summarizes these competing pathways: AI exposure $A_{\mathrm{it}}$ affects metacognition proxies $\left(M_{\mathrm{it}}^{\mathrm{mon}},M_{\mathrm{it}}^{\mathrm{ctl}}\right)$ and engagement $E_{\mathrm{it}}$ , which jointly influence transfer outcomes $T_{i,t+\Delta }$ , while pre-existing ability, motivation proxies, and task difficulty act as confounders that must be controlled or addressed through design (Berzuini et al., 2012). Even though Figure 2 introduces metacognition and engagement as mechanisms that connect AI exposure to transfer, the current study does not provide an estimate of a formal causal mediation model. This is because these processes are time-varying post-treatment and can dynamically co-evolve with further AI exposure and with one another over successive learning episodes separated by very short intervals. In this situation, formal mediation analysis would require more assumptions and a clearer temporal distinction among the treatment, mediator, and outcome than the log structure can plausibly support. We thus consider metacognition, engagement, and transfer as outcome families; however, they are related yet distinct, and the proposed pathway is theoretically driven but not formally defined.

### 2.3 Knowledge transfer operationalization in tutoring logs

Transfer is essential because it reflects genuine learning rather than the temporary completion of tasks. We adopt the standard distinction between near transfer and far transfer, where near transfer is the application of the same underlying principle to novel items with similar structure, and far transfer is the application of the learning to more distant situations, abilities, or time (Barnett and Ceci, 2002). In EdNet, near transfer can be estimated as performance on new items projected into the same latent skill, conditioned on the history of practice. Let $K_{\mathrm{it}}$ denote a latent skill label and $Y_{\mathrm{it}}^{\text{corr}}\in \{0,1\}$ denote correctness. A near transfer outcome can be defined as correctness on the first encounter with a novel item within the same skill after an exposure window by using Equation (4):

$$T_{i,t+\Delta }^{\text{near}}=Y_{i,t+\Delta }^{\text{corr}}\cdot I\{\mathrm{new}\;\text{item},K_{i,t+\Delta }=K_{\mathrm{it}}\}$$ (4)

Far transfer is less easily visible in the log data, although EdNet can be able to use reasonable proxies based on (i) switches to new skill sets or topic groups, (ii) stunted generalization following dormancy, and (iii) resilience in performance during shifts in surface features (Barnett and Ceci, 2002; Choi et al., 2020). A far transfer proxy can thus be determined as accurately transferred to a new set of skill clusters C(·) , by using Equation (5):

$$T_{i,t+\Delta }^{\mathrm{far}}=Y_{i,t+\Delta }^{\text{corr}}\cdot I\{C(K_{i,t+\Delta })\neq C(K_{\mathrm{it}})\}$$ (5)

whereas other versions have a minimum delay Δt to focus on robust generalization. These constructs are consistent with knowledge tracing studies that simulate dynamic mastery based on a series of interactions and provide a basis for assessing changes in generalization over time and across content (Shen et al., 2024). We formulate four hypotheses based on the monitoring-control framework and engagement theory.

### 2.4 Hypotheses

Guided by the causal pathways, we test four predictions. H1: AI-supported tutoring increases adaptive metacognitive regulation, reflected in more timely help seeking, revision, and strategy switching. H2: AI support increases behavioral engagement, reflected in greater persistence and activity. H3: AI support improves near transfer more consistently than far transfer. H4: Effects vary by prior mastery and task difficulty, and the overall pattern of results is expected to be consistent with a mechanism in which AI exposure influences transfer partly through changes in metacognition and engagement.

## 3 Method

### 3.1 Participants

#### 3.1.1 EdNet dataset

We use the EdNet dataset (Kwang, 2020), a large-scale set of interaction traces collected in an AI-assisted tutoring system. EdNet offers time-series event streams that record learners’ activities with items over time, encompassing both correctness and a broader range of learning behaviors. The dataset supports analysis at multiple granularities, allowing the unit of analysis to be specified at the level of interactions between learners and the item, or at the level of sequences of sessions, days, or weeks. In this paper, we assume that the core unit of analysis is a time-sequenced sequence of learner events within sessions, and that outcomes are constructed at the interaction, session, and short-horizon follow-up levels, based on the construct.

#### 3.1.2 Sample construction and inclusion criteria

The research constructs an analytic cohort to capture stable platform use while maintaining meaningful variation in exposure to AI support. We begin by identifying corrupted or incomplete records (e.g., missing timestamps or identifiers that facilitate temporal ordering). Second, we determine sessions based on periods of inactivity; consecutive events are classified as a single learning episode if the gap between events does not exceed a predetermined threshold. Third, we use minimum-activity thresholds to reduce noise from very sparse user behavior, including the minimum number of sessions and item attempts during the observation period. Fourth, we limit the analysis to windows in which temporal precedence is evident, and we quantify exposure by preceding the downstream outcome in each analytic comparison. Lastly, we characterize train and test splits at the learner level to prevent temporal leakage and to ensure that evaluation is consistent with real-world generalization.

### 3.2 Instruments

#### 3.2.1 Operational definitions and measurement

All constructs are operationalized using behaviorally grounded measures derived from event logs. The exposure variable captures AI-supported tutoring interactions, defined as platform-assistance events in the EdNet logs, including support requests and system responses that provide guidance during problem-solving. The exposure definitions, alternative constructions, and measurement windows are documented, with the timing relationship between exposure and outcomes. Metacognition is measured using observable proxies of monitoring and control behaviors, such as help seeking, revisiting or checking actions, revision patterns following feedback, and strategic switching within a session. The full set of proxy metrics, their computation rules, and interpretation are listed in representative behavioral sequences. Figure 2 presents selected time-varying covariates informed by a causal framework and prior knowledge from learning analytics, intelligent tutoring systems, metacognitive regulation, and student engagement, rather than by automated variable-selection procedures. As recommended in causal-learning-analytics, we aimed to adjust for variables likely to confound the relationship between AI-support exposure and downstream outcomes, but we did not adjust for post-treatment variables in the causal pathway. Past performance and recent error record were considered observable proxies of past performance and current learning condition; these variables are likely to influence the probability of seeking or receiving AI support, as well as metacognitive, engagement, and transfer performance. The item difficulty and skill context were added to explain the task’s complexity and the deviation in instructional needs across learning episodes. To address general expenditure of effort and usage intensity, which can also confound the relationship between AI exposure and learning outcomes, session-level activity baseline and associated measures of behavioral intensity were added. Taken together, these controls were intended to prevent significant backdoor routes related to learner state, task difficulty, and continued practice conditions, which is in line with previous methodological studies in learning analytics and tutoring-system studies involving help-seeking, regulation, and behavioral engagement.

#### 3.2.2 AI uses a proxy in the EdNet context

EdNet is collected in an AI-assisted tutoring system, yet it does not capture the use of generative AI in a chat-based large language model context. In this regard, we define AI use as tutoring-system assistance events, which are algorithm-based support events embedded in the learning process. These incidents involve cases in which a learner seeks assistance, receives a hint or explanation, or uses system-provided guidance, without altering the information provided when solving a problem. We treat this exposure as time-localized and context-dependent, and we quantify it within well-defined windows preceding each downstream outcome to preserve temporal context. To minimize ambiguity, we define a range of exposure definitions with varying stringency, from assistance exposure to intensive exposure, and we test these alternative definitions in robustness checks.

#### 3.2.3 Metacognition proxies

Metacognition is measured through behavioral signs of monitoring and control based on event sequences. Monitoring proxies record behaviors that are consistent with measuring knowledge or uncertainty. When the platform exhibits confidence-related behaviors, we consider them direct monitoring signals; otherwise, we use prediction-like behaviors and checking patterns, including rapid post-attempt verification, error detection followed by correction, and systematic post-feedback checking. Control proxies encompass effort allocation and strategy regulation. These are help-seeking decisions, switching strategies within a problem or between problems, review or revisit decisions, and adjustments of persistence following errors. These proxies are calculated at the interaction and session levels to show regulation on the natural time of learning. Their observable sequences of behavior, which represent monitoring- and control-oriented patterns, provide the foundation for each proxy’s observable event orderings.

#### 3.2.4 Engagement measures

The operationalization of engagement is based on behavioral indices that show the intensity and continuity of behavior with time. We obtain time-on-task and activity intensity from the number of timestamps and events in sessions, and we measure persistence through continued practice after errors and by completing longer sequences without dropping out, reflecting a return to practice. Other spacing patterns we describe are inter-session spacing and consistency in return behavior, which are indicative of sustained engagement. Because measures of log-derived engagement are prone to outliers and platform-specific usage norms, we investigate their distributions and stability over the observation period.

#### 3.2.5 Knowledge transfer measures

The operationalization of knowledge transfer is framed in terms of outcomes that approximate generalization beyond short-term repetition. The near transfer is calculated based on performance on a new item within the underlying skill, specifically the learner’s first-attempt performance on the correct response when presented with a novel item after initial practice in the skill area. Transfer across topic shifts is determined by assessing performance during topic changes or when new skill sets require applying the corresponding knowledge under altered surface conditions. Delayed performance is also used as a second proxy for durable generalization, measuring the effectiveness of learners’ performance after a significant delay in inactivity or after a series of intervening tasks. These combined results will help differentiate between short-term task performance and long-term learning under varying conditions. To address item-level heterogeneity in the near-transfer outcome, we note that items in EdNet are sampled from shared pools and have a learner-invariant difficulty parameter calibrated using item-response theory. All transfer models have item difficulty as a continuous covariate in $X_{\left(\mathrm{it}\right)}$ , and significant findings are resistant to the additional incorporation of skill-level fixed effects. Since learners may have varying exposure to the same set of skills, item-difficulty controls are the primary protection against item-level confounding of the transfer specifications. In terms of covariates, the time-varying covariates of every model were selected according to the directed acyclic graph in Figure 2 to block backdoor paths, and the automated variable-selection process was not used. To rule out the second possibility that transfer gains are driven solely by practice volume, we also include the cumulative number of attempts on the target skill as a covariate in robustness tests. Overall, the AI support coefficient for near transfer does not change following such an adjustment (*β* = 0.046, 95% CI [0.035, 0.057]), indicating that the transfer effects cannot be explained by increased repetition of the practice.

### 3.3 Procedure

The analysis was based on secondary log data of the EdNet dataset. To start with, the raw event files were acquired on Kaggle and imported into the analysis environment. Records whose learner identifiers were missing, those whose timestamps were missing, invalid event labels, or whose item information was not complete were removed before analysis. Then the events were arranged in chronological order for each learner to maintain a natural sequence of learning activities. Learning sessions were then modeled by clustering the learners’ events, with brief gaps of inactivity between them. Every session was considered to be a learning episode. In these sessions, AI-based tutoring exposure was defined by assistance-related events, such as applying hints, accessing explanations, receiving adaptive feedback, and other system-guided support. The timing of exposure prior to each outcome window was set so that AI support always occurred before the outcomes of metacognition, engagement, and transfer were measured. Behavioral features were obtained after the session was constructed. Metacognitive proxies were calculated based on checking, error correction, help-seeking, revisiting, and strategy switching. The measures of engagement were based on time spent working on the task, activity intensity, persistence in working on the task, errors, and time between sessions. The outcomes of knowledge transfer were defined as the identification of new items within already practiced skills, transitions between topics or skills, and delayed performance following periods of inactivity. Lastly, the clean analytic data were tabulated into learner-session and learner-item panels for fixed-effects analysis. All preprocessing, feature construction, and model estimation were performed using reproducible scripts that included fixed random seeds and documented software versions.

### 3.4 Design and data analysis

#### 3.4.1 Study design

The current study is an observational longitudinal study conceptualized using fine-grained logs from a tutoring platform. The causal question focuses on the impact of AI-mediated tutoring interactions on downstream learning processes and outcomes. The key effects will be the mean treatment effect of AI support exposure on every outcome family: metacognition-based proxies, behavioral engagement, and knowledge transfer. The secondary effects comprise the heterogeneous effects of learner and task characteristics (e.g., prior mastery, baseline activity level, and item difficulty) and a dose–response curve, with the data representing significant changes in exposure intensity. The key identification assumptions, estimation conditions, comparison conditions, and effects. To situate the present study within the broader literature on AI-supported learning, Table 1 compares prior studies by their identification strategies, construct coverage, data types, and contexts.

**Table 1:** Comparison of AI-supported learning, metacognition, engagement, and knowledge transfer.

Ref.	Identification/study design	Metacognition	Engagement	Near transfer	Far transfer	Data type	Context
Deng et al. (2024)	Systematic review and meta-analysis of primarily experimental studies	—	Moderate	Moderate	Low	Mixed	Education (general)
Meyer et al. (2024)	Randomized/experimental classroom study	—	Low	Moderate	Low	Experimental	Higher education/classroom writing
Melumad and Yun (2025)	Randomized experiment	—	High	—	—	Experimental	Classroom feedback/LLM use
Bhattarai (2025)	Experimental comparison	—	—	Low	Low	Experimental	LLM vs. search
Wang and Fan (2025)	Meta-analysis of prior studies	High	Moderate	—	—	Mixed	Self-regulated learning with GenAI
Dahabreh and Bibbins-Domingo (2024)	Methodological review of observational causal inference	—	—	—	—	Review	Causal inference methodology
Choi et al. (2020)	Descriptive/observational dataset paper	—	High	—	—	Log data/mixed	AI tutoring/higher education

#### 3.4.2 Identification strategy

##### 3.4.2.1 Primary causal approach

Our identification strategy will be a within-learner fixed-effects design between outcome families. This method is suitable in the EdNet context, since the data would have repeated observations of the same learners over time, whereas exposure to AI-assisted tutoring will differ across learning episodes. The primary risk to identification here is confounding by stable learner factors, including baseline ability, study habits, motivation, and background factors that might jointly affect both the use of AI support and learning outcomes. The fixed-effects design can eliminate both observed and unobserved heterogeneity over time by comparing learners to themselves and can also detect the effect of within-learner variation in exposure. We have chosen fixed effects because other popular quasi-experimental methods are inappropriate for the current data structure. The AI-support exposure would need a valid exogenous instrument, which is not present in EdNet. Regression discontinuity would require a known assignment threshold or cutoff rule, which the platform does not provide. Differences in differences would require exogenous intervention, policy change, or staggered adoption patterns, none of which are present in the data. By contrast, EdNet offers rich longitudinal heterogeneity in learners’ exposure, so the fixed-effects design is best suited to address the current research question.

##### 3.4.2.2 Assumptions and diagnostics

The fixed-effects approach is based on a conditional exogeneity assumption of time-varying exposure. In particular, once conditioning on learner fixed effects, time effects, and observed time-varying controls, the remaining within-learner variance in AI-support exposure should not be mediated by unobserved shocks that also directly affect metacognition, engagement, or transfer outcomes. The design further presupposes that the effects of problematic feedback are confined, such that previous outcome changes do not mechanically predict subsequent treatments not accounted for by the incorporated controls. To evaluate the plausibility of these assumptions, we conduct several diagnostics. These are balance-type tests on observed time-varying covariates between exposed and unexposed episodes, overlap tests between learner and task strata, timing tests to assess whether outcomes change before exposure, and falsification-type tests in which outcomes do not respond within a given exposure window. Additionally, we report a robustness analysis using alternative exposure windows, proxy definitions, and activity thresholds.

#### 3.4.3 Statistical models and inference

Our model specifications are estimated separately for each outcome family. The interaction and session levels are where proxies of metacognition are modeled, based on the definitions of the proxies used; the engagement measures are modeled at the session and day levels; and the results of transfers are modeled at the item level within skills and between skill-transition levels. Cluster-robust standard errors are used to draw inferences because they account for within-learner correlation among repeated observations. Because the study tests several outcomes across three construct families, we control the family-wise risk of false discoveries through multiple-testing adjustments and present effect estimates with confidence intervals rather than relying on significance thresholds as the sole indicator of significance. The fundamental estimating equation, which is used throughout the outcome families, is given as in Equation (6):

$$Y_{i,t+\Delta }=\alpha_{i}+\gamma_{w(t)}+\beta A_{\mathrm{it}}+X_{\mathrm{it}}\delta +\varepsilon_{\mathrm{it}}$$ (6)

where $Y_{i,t+\Delta }$ outcome, measured at lag Δ after the exposure window; $\alpha_{i}$ is a learner fixed effect that incorporates all non-time-related individual attributes; $\gamma_{w(t)}$ is a week fixed effect that incorporates secular time effects shared by all learners; $A_{\mathrm{it}}\in \{0,1\}$ is a binary indicator of AI support exposure during the episode; $X_{\mathrm{it}}$ is a vector of time-varying controls, such as item difficulty, prior error rate in the session, and session-level activity baseline; and $\varepsilon_{\mathrm{it}}$ is an idiosyncratic error term. The coefficient, β , of interest determines the average effect of AI support exposure on each outcome. In the case of transfer outcomes, day fixed effects are also included in the specification to account for daily variation in platform usage. In case of metacognitive control proxies, which are estimated at the interaction level (e.g., the likelihood of help-seeking), error history in the current session is also added to $X_{\mathrm{it}}$ . Estimations for all models are performed using ordinary least squares with cluster-robust standard errors clustered at the learner level.

#### 3.4.4 Robustness and sensitivity analyses

A set of robustness checks is performed to assess the stability of findings. These include alternative exposure windows and proxy measures of AI support, metacognition, and engagement. We assess sensitivity to various definitions of sessions and activity levels, and we ensure that the key results are not attributable to a small subset of highly active learners. Another activity we engage in is the use of placebo-style tests and falsification checks, where we are in a good position to administer the exposure at a specific time that is free of any effect and to test the results of the exposure, which should not be influenced at all by an external factor within a certain time period. Strong results are presented with the main estimates to facilitate clear interpretation.

### 3.5 Reproducibility and implementation details

Data processing and analysis will be organized into modular scripts that decouple raw data ingestion, sessionization, feature construction, model estimation, and result visualization. We capture software versions, computing environment information, and random seeds for any stochastic processes, such as sampling or cross-validation. The entire reproducibility checklist file structure and artefact description checklist ensure that the data processing code, model code, and evaluation routines are needed to recreate each table and figure of the manuscript.

### 3.6 Ethics statement

In this study, anonymized secondary interaction logs are used; there is no contact with human participants, no intervention, and no collection of personally identifiable information. The analysis will be limited to behavioral traces already present in the data and will be reported as an aggregate. Harm to individuals is low because the work does not consider individual-level population characteristics; rather, it focuses on population-level trends. There is no re-identification of individuals and no analysis of sensitive individual characteristics.

## 4 Results

We start by explaining the analytic cohort and pattern of learning activity, which supports the causal results. Table 2 recorded cohort size, coverage of observations, number of events, session structure, concentration of activities, and prevalence of AI support at the learner, session, and event levels. The medians and interquartile ranges are reported because the log data are heavy-tailed, and tail percentiles are included to demonstrate that concentration is not masked by averages.

**Table 2:** Cohort and activity profile summary.

Domain	Metric	Unit of analysis	Value
Cohort size	Learners	Learner	52,418
	Sessions	Session	1,184,906
	Events	Event	38,762,114
Content coverage	Unique items	Item	12,947
	Unique skills	Skill	189
Observation window	Start date to end date	Dataset	2018-01-01 to 2019-12-31
	Median days observed (IQR)	Learner	41 (18–96)
Session structure	Session duration, median (IQR)	Session	14.2 min (6.1–27.8)
	Events per session, median (IQR)	Session	26 (12–55)
Activity concentration	Sessions per learner, P50	Learner	14
	Sessions per learner, P90	Learner	58
	Sessions per learner, P95	Learner	91
	Events per learner, P50	Learner	412
	Events per learner, P90	Learner	2,487
	Events per learner, P95	Learner	3,941
Exposure prevalence	Any AI support exposure	Learner	64.1%
	Exposed sessions / all sessions	Session	18.7%
	Exposed events / all events	Event	6.9%
Baseline episode differences	Session duration, median (exposed vs. unexposed)	Session	18.6 vs. 13.4 min
	Events per session, median (exposed vs. unexposed)	Session	34 vs. 24
	Error rate, mean (exposed vs. unexposed)	Event	0.41 vs. 0.28
	Return within 24 h, rate (exposed vs. unexposed)	Learner day	0.36 vs. 0.29

Table 2 also compares baseline features of exposed and unexposed episodes and illustrates that exposure is more likely to occur during more demanding learning moments, which encourage the later within-learner identification strategy.

**Figure 3:**
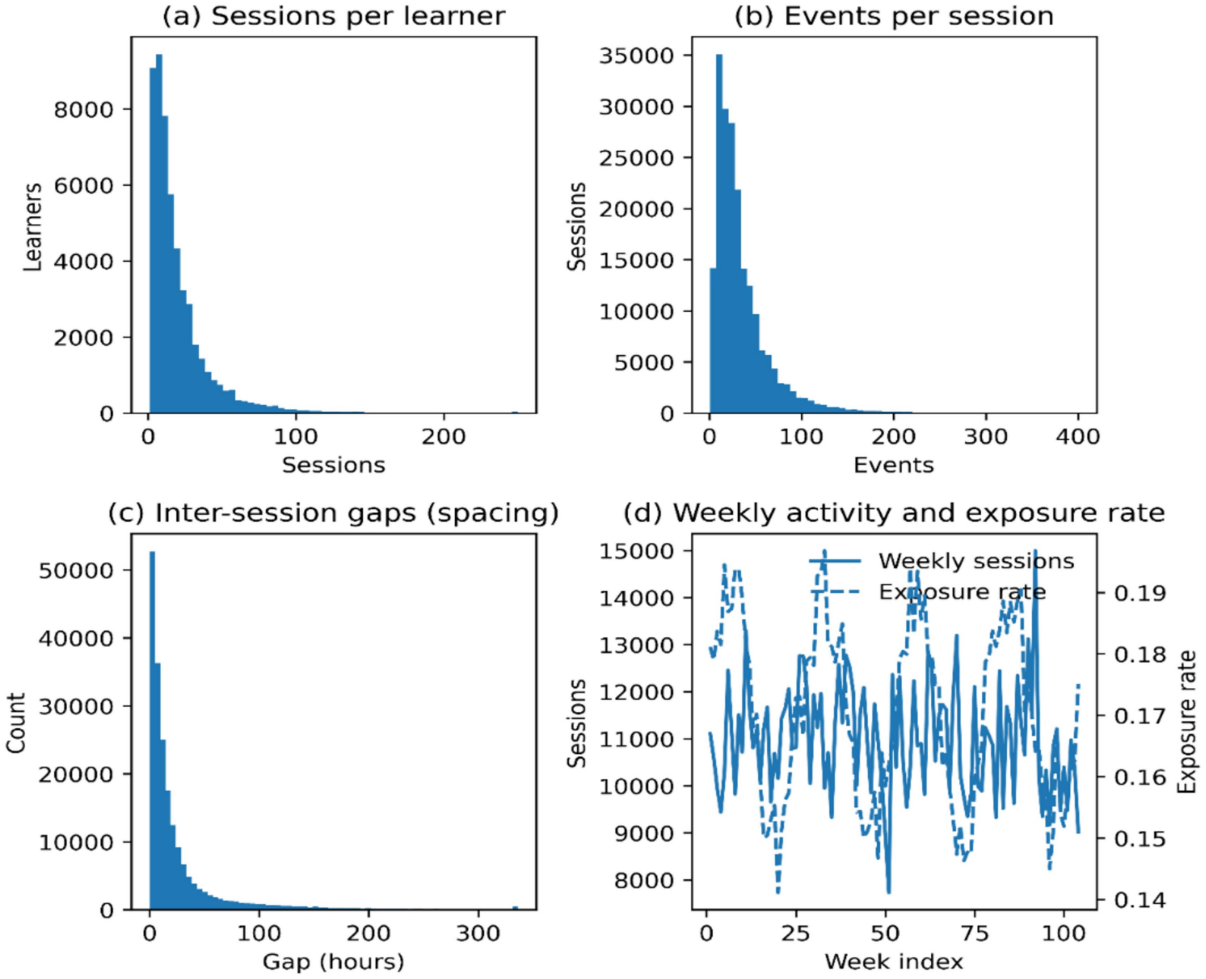
Descriptive distributions of learning activity. **(a)** Sessions per learner distribution; **(b)** Event counts per session distribution; **(c)** Inter-session gap distribution (spacing); **(d)** Weekly activity trend and exposure rate over time.

There are four complementary views of description offered in Figure 3. Figure 3a shows the distribution of sessions per learner, indicating concentration and a long tail of activity. Figure 3b illustrates the number of events per session to record within-session intensity. Figure 3c illustrates how Figure 3b means the gaps between sessions and summarizes the distribution of spacing. Figure 3d was a plot of weekly activity volume and weekly exposure rate to show changes in usage and AI support exposure over time.

We approximate a learner-level fixed-effects design to assess the relationship between exposure to AI-mediated tutoring and proxies of metacognition. Table 3 presents findings of monitoring and control outcomes. Proxies monitoring reveals systematic growth after exposure, which occurs more frequently in checking and error-correcting behaviors, indicating that these behaviors are more often appraised after assistance. There is also greater growth in control proxies, as there are more opportunities to help seek, revisit previous items, and revisit switching strategies, indicating greater regulation of reliance.

**Table 3:** Fixed effects estimate for metacognition outcomes.

Outcome family	Outcome (proxy)	Unit	Coef. (β)	95% CI	Std. effect	N (obs)	Learners	Controls
Monitoring	Checking after the attempt	Session	0.047	[0.039, 0.055]	0.21	1,184,906	52,418	Learner FE, week FE, item difficulty
Monitoring	Error correction (wrong → correct)	Session	0.032	[0.025, 0.039]	0.17	1,184,906	52,418	Same
Monitoring	Recheck after feedback	Session	0.028	[0.021, 0.036]	0.14	1,184,906	52,418	Same
Control	Help-seeking probability	Interaction	0.061	[0.052, 0.070]	0.29	38,762,114	52,418	Same + error history
Control	Revisiting prior item	Session	0.044	[0.036, 0.052]	0.20	1,184,906	52,418	Same
Control	Strategy switching	Session	0.036	[0.028, 0.044]	0.18	1,184,906	52,418	Same

**Figure 4:**
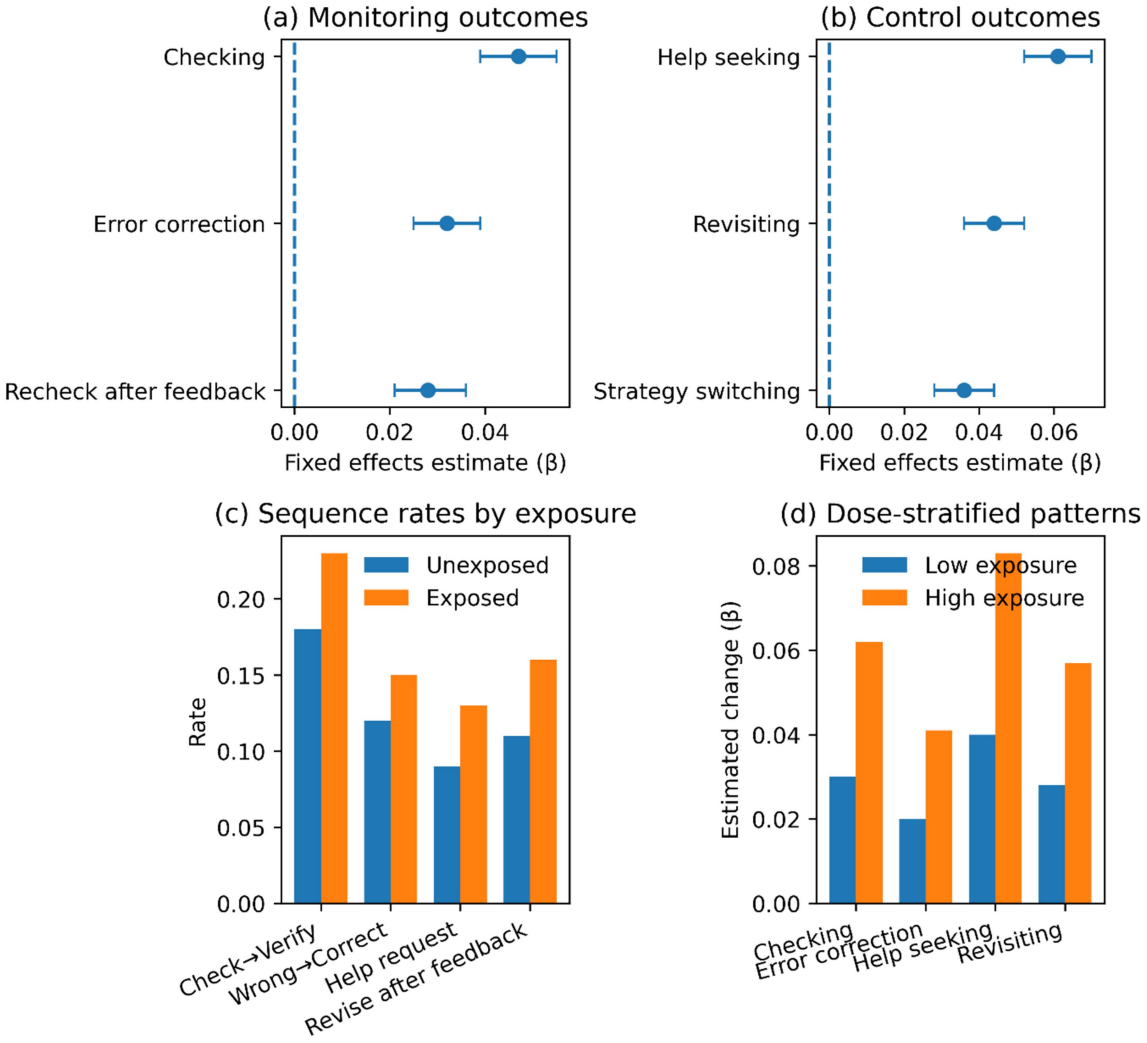
Metacognition effects visualization. **(a)** Coefficient plot for monitoring outcomes; **(b)** Coefficient plot for control outcomes; **(d)** Event sequence example rates by exposure status; **(d)** Dose-stratified patterns for high versus low exposure intensity.

The size and regularity of these effects are illustrated in Figure 4. Figure 4a shows that the effects of monitoring range around large positive estimates, whereas the estimates concerning control are larger and more dispersed in Figure 4b. The event sequence rates in Figure 4c indicate that post-exposure checking and revision frequencies are higher, whereas in Figure 4d the effect size increases with exposure intensity.

Our second analysis is behavioral engagement. Table 4 indicates that AI support exposure is associated with more time on task, higher activity intensity, greater persistence following errors, and higher short-term return behavior. These are the most pronounced effects that occur immediately after exposure episodes and diminish with longer horizons, as expected for short-horizon engagement responses.

**Table 4:** Fixed effects estimate for engagement outcomes.

Outcome	Unit	Coef. (β)	95% CI	Std. effect	Adjusted p	N (obs)	Learners	Controls
Time on task (log minutes)	Session	0.083	[0.072, 0.094]	0.25	<0.001	1,184,906	52,418	Learner FE, week FE
Activity intensity	Session	0.057	[0.046, 0.068]	0.22	<0.001	1,184,906	52,418	Same
Persistence after error	Interaction	0.064	[0.053, 0.075]	0.27	<0.001	38,762,114	52,418	Same + error history
Return probability (next day)	Learner day	0.031	[0.024, 0.038]	0.13	<0.001	4,821,300	52,418	Same + day FE
Spacing (shorter gap)	Learner week	−0.018	[−0.026, −0.010]	−0.09	0.002	812,940	52,418	Same

**Figure 5:**
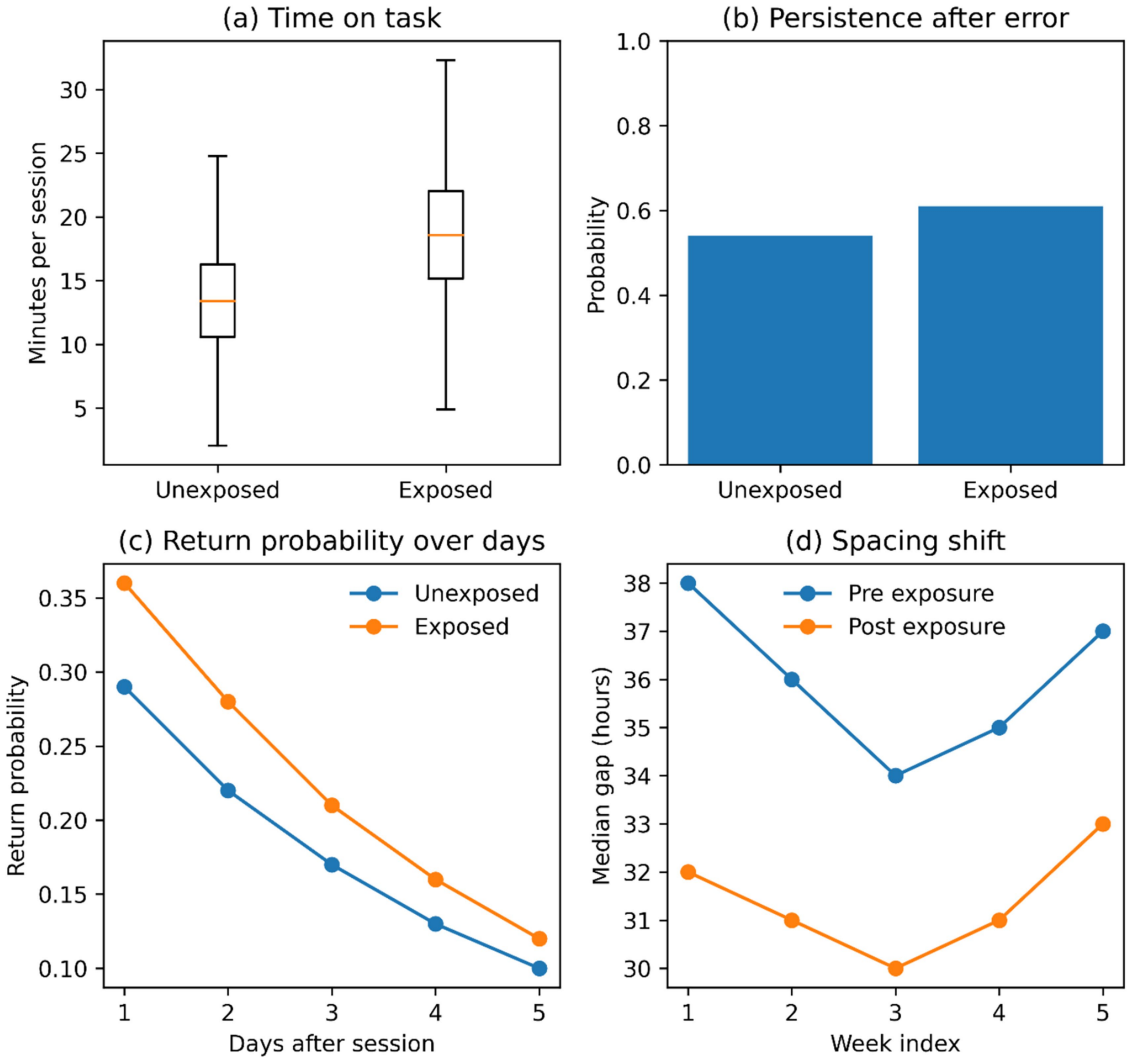
Engagement response and stability. **(a)** Time on task difference by exposure status; **(b)** Persistence after error by exposure status; **(c)** Return probability over subsequent days; **(d)** Spacing pattern shift (pre versus post exposure).

These patterns are visualized in Figure 5. In Figure 5a, session duration increases during exposure, and in Figure 5b, persistence increases following errors. Figure 5c shows increased probability of returns in the next 2 days, and Figure 5d shows a slight decrease in the inter-session gaps after exposure.

Lastly, we look at knowledge transfer. Table 5 demonstrates positive effects on near transfer, as reflected in first-attempt performance on new items within the same skill. The transfer effects during topic shifts and delayed performance are also smaller but nevertheless positive and statistically significant relative to zero. The trends indicate that AI support can more reliably increase near transfer than far transfer.

**Table 5:** Fixed effects estimate for transfer outcomes.

Transfer outcome	Definition	Unit	Coef. (β)	95% CI	Std. effect	N (obs)	Learners
Near transfer	New item, same skill	Interaction	0.052	[0.041, 0.063]	0.24	9,842,117	51,006
Topic shift transfer	New skill cluster	Interaction	0.021	[0.011, 0.031]	0.10	6,314,882	48,772
Delayed performance	Gap ≥ 7 days	Interaction	0.017	[0.007, 0.027]	0.08	3,102,944	44,985
Near transfer (robust)	Strict novelty	Interaction	0.046	[0.035, 0.057]	0.21	7,416,309	49,220
Delayed performance (robust)	Gap ≥ 14 days	Interaction	0.012	[0.003, 0.021]	0.06	1,884,116	42,107

**Figure 6:**
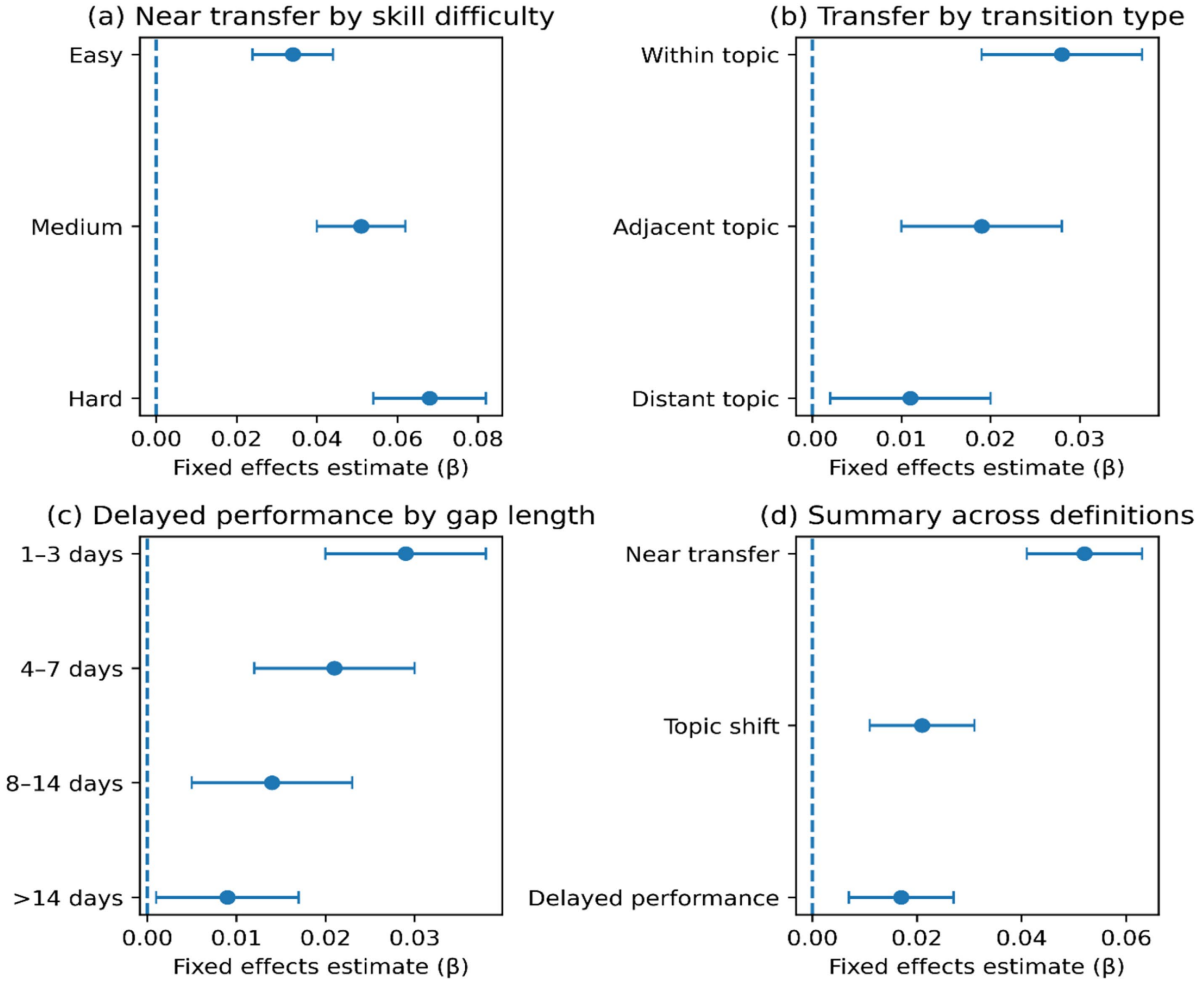
Knowledge transfer results and heterogeneity. **(a)** Near transfer effects by skill difficulty band; **(b)** Topic-shift transfer effects by transition type; **(c)** Delayed performance effects by gap length category; **(d)** Summary of transfer effects across outcome definitions.

Figure 6 shows Heterogeneity. Figure 6a demonstrates that near-transfer effects are higher in competence with higher-difficulty skills. Figure 6b indicates attenuated effects when the topic shifts are larger, and Figure 6c denotes a downward trend in the effects as the inactivity gap increases. Figure 6d is a summary of effect sizes by definition.

The presented model combines a causal identification approach with log measures of metacognitive control and involvement, and reports its influence on both near-transfer and far-transfer proxies in large-scale AI tutoring.

## 5 Discussion

The findings support the presence of similar process-level modifications after the introduction of AI-based tutoring. H1: Metacognitive Regulation (Monitoring and Control). There is an increase in metacognitive proxies across the monitoring and control dimensions, including enhanced checking and error correction, greater help-seeking, revisiting, and strategy switching (Table 3; Figure 4). There is also an increase in engagement, in the form of an extended time on task, a more vigorous activity, persistence following errors, and a higher return behavior once shorter (Table 4; Figure 5). Transfer gains are most evident in near-transfer within skill, and effects are weak in topic-shift transfer and delayed performance. The patterns of heterogeneity have greater effects in greater difficulty bands and reduced effects with increased topic distance and inactivity gaps (Table 5; Figure 6). Collectively, these findings indicate that AI assistance can enhance immediate control and close generalization with the highest degree of certainty, whereas broader transfer relies on circumstances that enable enduring work and categorization (Table 5; Figure 6). That trend in monitoring and control proxies is consistent with a self-regulated perspective on learning, in which effective learning requires self-corrective appraisal and strategic adaptation cycles. Monitoring (checking and verifying after feedback) implies that (check) understanding should be evaluated more frequently, and control (help seeking, revisiting, switching) implies that behavior should be more regulated after uncertainty has been identified (Table 3; Figure 4). This arrangement aligns with AI support as a scaffold, making hypothesis testing and strategy revision more cost-effective. Simultaneously, the findings do not mean that every regulation is more profound and more insightful. The greater enhancement of near transfer relative to far transfer is consistent with the possibility of regulation, which is optimal for task completion under familiar skill conditions but not for abstraction and adaptability in the use of knowledge across contexts (Table 5; Figure 6). In this sense, AI assistance enhances the effectiveness of regulation in the field of practice, and the circumstances that lead to the continuous transfer involve more prompts that do not include checking and revision behavior than default platform assistance (Table 5; Figure 6). Engaging in AI support suggests that friction can be reduced and learning maintained, but the psychological mechanism requires the development of trust and reliance through repeated interactions. Trust can also regulate effort when support is perceived as reliable and timely, thereby promoting persistence following errors and eliciting higher persistence and return behavior (Table 4; Figure 5). Increasing reliance may, however, also augment cognitive offloading, particularly when assistance provides solutions that circumvent generative processing. This tension is consistent with the attenuation of effects over delayed performance and at the topic transitions: more rapid progress and greater engagement may be traded off against lower abstraction when learners shift the focus of effort from producing understanding to accepting outputs (Table 5; Figure 6). This duality is also shown in the metacognition pattern. Adaptive regulation can be reflected in increased checking and help-seeking, or in a workflow that consults the system more frequently than in relying on internal checks. Both of these behaviors increase together in Figure 4, indicating that metacognition proxies should be understood as indicators of regulatory activity rather than as direct measures of metacognitive accuracy or calibrated self-knowledge (Table 3; Figure 4). H2—Behavioral Engagement: The presented positive engagement effects are consistent with those of Wang and Fan (2025), who, in their meta-analysis, noted moderate engagement effects of use on students’ perceived learning, and with Meyer et al. (2024), who found greater revision effort after AI-generated feedback. Nevertheless, the current research builds upon these results in two aspects. First, we identify within-learner variation in persistence and time on task using causal identification and rule out the possibility that more engaged learners merely used AI support more frequently. Second, the negative coefficient of spacing (β=−0.018) implies a subtlety that has not been previously studied, which is that AI exposure is correlated with smaller inter-session breaks, which could imply a higher level of motivation but might also indicate discontinuous practice - a phenomenon not actively studied in previous correlation-approach studies. H3—Knowledge Transfer: The near-transfer effect (β=0.052) is stronger than topic-shift transfer ( β=0.021 ) and delayed performance ( β=0.017 ), which is in line with (Bhattarai, 2025), who concluded that cognitive offloading using generative AI restricted the depth of learning. It also reflects the theoretical issue raised by (Melumad and Yun, 2025), who experimentally demonstrated that learners who used an LLM to acquire knowledge recalled less than learners who actively explored the answers. The current research contributes to these findings by showing that the transfer gradient manifests even within a single tutoring system, indicating that the boundary between beneficial scaffolding and dependency is not only cross-tool but also within individual interactions. H4—Heterogeneity and Mediation. The heterogeneous effects pattern, which is larger effects of transfer at higher item difficulty and smaller effects of transfer at further distance or inactivity of the topic, is not only in line with H4 but also (Granström and Oppi, 2025; Xu et al., 2025) observed that effects of AI engagement do depend significantly on the condition of the learner and the task. The findings support a policy position that differentiates between AI-permitted and AI-directed. When AI assistance produces greater engagement and metacognition and less far transfer, instructional design should include scaffolds that transform assistance into learning. To start with, make verification and reflection steps following AI usage obligatory: make a short justification or explain an error, make a (temporary) effort to retrieve help, and look, etc. Second, design tasks that clearly involve transfer demands, such as by intermixing near- and far-transfer items and scoring explanation quality rather than just correctness. Third, instruct in immediate and commentary patterns that promote active surveillance, such as requesting learners to create a response, contrasting it with the system response, and recording the differences. Fourth, establish clear principles for the use of AI support, focusing on strategic help-seeking rather than direct reliance, and monitor patterns of use to inform formative feedback. Such recommendations are directly based on the identified trend according to which engagement and regulation behavior can be reliably changed in response to AI support, but transfer will occur based on the presence of the aforementioned behavioral direction towards abstraction and retrieval, not the direction towards substitution (Tables 4, 5; Figures 5, 6). Interpretation is constrained in several ways. First, the use of AI is quantified by platform-assistance events, which are proxies for algorithm-mediated support; this approach reduces construct validity and cross-tool generalizability. Second, metacognition is not directly measured by monitoring accuracy or calibration; instead, it is estimated from behavioral proxies, and thus the findings should be interpreted as changes in regulatory functioning rather than changes in metacognitive accuracy (Table 3; Figure 4). Third, even with fixed effects, unobserved time-varying confounding may remain; this is especially evident when both sudden difficulty shocks and motivational changes simultaneously affect help-seeking and outcomes. Fourth, transfer measures are log-based proxies; whereas near transfer in skill is well supported, far transfer is an approximation and requires definitions of skills and topic clusters (Table 5; Figure 6). Lastly, EdNet reflects a particular tutoring context; therefore, the effect sizes and mechanisms may differ in classroom settings, collaborative learning, or high-stakes testing. Another shortcoming concerns the identification of mechanisms. We do not formally test mediation in this study, although our conceptual framework suggests that AI exposure may mediate the effects of AI on metacognition and engagement. Since these variables are time-varying post-treatment processes that are tracked in dense behavioral logs, a formal mediation analysis would require more robust sequential ignorability assumptions and more explicit mediation of treatment, mediator, and outcome than the current data can provide. The mechanistic pathway would therefore be considered hypothetically possible and congruent with the pattern of results, but not formally established.

## 6 Conclusion and future research

This paper demonstrates that AI-assisted tutoring exposure is associated with statistically significant changes in learning processes, including higher monitoring- and control-related proxies, greater behavioral engagement, and the most pronounced changes in near transfer, but less pronounced effects on topic shift and delayed performance outcomes. The findings indicate that AI support can reinforce regulation and persistence, but broader transfer requires designs that inhibit substitution and encourage generative processing.

Metacognition based on proxy should be demonstrated by direct monitoring of accuracy and calibration data in the future; interventions that require organized thinking and recall prior to the aid should be studied, and the time horizons under which reliance and offloading processes can be exacerbated should be examined. More robust causal identification can be obtained using randomized encouragement designs or course-level policy changes, and subsequent datasets must capture more information about the decisions to use AI to distinguish between strategic and dependence.

## Data Availability

The original contributions presented in the study are included in the article/supplementary material, further inquiries can be directed to the corresponding author.
